# Identification of Risk Areas for *Gloydius* Snakebites in South Korea

**DOI:** 10.3390/ani13121959

**Published:** 2023-06-12

**Authors:** Youngjoo Moon, Chaewan Kim, Sungsoo Yoon, Wanmo Kang

**Affiliations:** 1 Department of Forest Resources, Graduate School, Kookmin University, 77 Jeongneung-ro, Seongbuk-gu, Seoul 02707, Republic of Korea; yjmoon@kookmin.ac.kr (Y.M.); cwkim@kookmin.ac.kr (C.K.); 2 Ecological Information Team, National Institute of Ecology, 1210 Geumgang-ro, Seocheon-gun 33657, Republic of Korea; yssfran@nie.re.kr; 3 Department of Forest Environment and Systems, College of Science and Technology, Kookmin University, 77 Jeongneung-ro, Seongbuk-gu, Seoul 02707, Republic of Korea

**Keywords:** *Gloydius* snake, hiking trail, random forest, species distribution model, venomous snake

## Abstract

**Simple Summary:**

Efforts to prevent snakebites on hiking trails are crucial because such accidents can lead to fatalities, particularly in isolated areas. This study developed random forest species distribution models to predict the potential habitats of *Gloydius* spp. in South Korea, verified a significant relationship between the habitat distribution and actual snakebite accidents at the regional level, and assessed snakebite risks on trails in the national parks of Korea. The model identified high-risk snakebite areas, which will help to develop preventative solutions for hikers’ safety.

**Abstract:**

Snakebites can pose a significant threat to human health as the destruction of natural habitats and increased human intrusion into ecosystems result in more frequent encounters with snakes. Mitigation measures for snakebites are particularly crucial for hiking trails where transportation of snakebite victims to medical facilities is challenging due to limited emergency resources and difficult access. This study employed a random forest-based species distribution model approach to investigate the potential habitats of *Gloydius* spp., specifically *Gloydius saxatilis*, *Gloydius brevicaudus*, and *Gloydius ussuriensis*, in South Korea and to assess the snakebite risk in national parks. Potential habitats of *Gloydius* spp. were identified and visualized by overlaying binary maps derived from species distribution models (SDMs) of each *Gloydius* spp. that corresponded to high-risk snakebite areas. In addition, hiking trails with high snakebite risk in the national parks were identified after demonstrating the statistical correlation between the potential habitat distribution of *Gloydius* spp. and the actual snakebite incidents in major regions of South Korea. The primary environmental variables determining *Gloydius* spp. habitat were the topographic position index, slope, and the annual average of the maximum and minimum temperatures. The potential habitat of *G. saxatilis* generally appeared in high-altitude mountainous areas, mostly in the eastern part of the study area. Favorable habitats for *G. brevicaudus* and *G. ussuriensis* were predominantly located in mountainous areas throughout the study area, with the exception of some high-altitude mountainous terrain in the east. The number of snakebite incidents per 10,000 people was significantly correlated with the area ratio of *Gloydius* spp. potential habitat (Spearman’s rho = 0.638, *p* < 0.01). The proportion of snakebite risk areas among national parks in South Korea ranged from 18% to 57%. This study can support practical solutions to prevent injuries and fatalities among hikers due to snakebites by identifying areas with a high risk of snakebite accidents at the hiking-trail level.

## 1. Introduction

The increase in human interference with, and destruction of, ecosystems has been blurring boundaries between human settlements and natural ecosystems [1], resulting in a rise in injuries and fatality accidents associated with wildlife encounters [2,3]. In particular, snakes and humans have had encounters since ancient eras, as evidenced by historical records of snake-induced fatal accidents [4]. Snakes, which include diverse species that are distributed worldwide except for in extreme climates [5], cause more than 5 million cases of snakebite incidents annually [6]. The expansion of human settlements, driven by urbanization and accompanied by snake habitat relocation and destruction, can increase the likelihood of human casualties by venomous snakebites as well as the frequency of snake encounters [7,8]. Predicting the potential habitats of wild snakes is crucial for understanding snake encounters, identifying patterns of snakebite incidents, and reducing the number of snakebite victims.

Of the more than 3000 known snake species, approximately 200 species (i.e., 15%) are considered dangerous because their bites can be fatal [9,10]. Bites from venomous snakes can cause a wide range of symptoms, from mild symptoms such as respiratory difficulties, headaches, and speech impairment, to severe symptoms including muscle, kidney, and organ damage, potentially leading to death [11,12]. Some venomous snakes tend to ambush from rock crevices, tree roots, or low shrubs, resulting in unexpected snakebite incidents, particularly in mountainous terrain with various landscape features [5,13,14,15]. In dense forests, the transportation of snakebite victims to medical facilities is not only difficult due to the complex terrain, but also challenging because of insufficient emergency resources for handling venomous snakebite accidents in the vicinity [16,17]. Therefore, strategies for assessing the risk of venomous snakebite accidents in forests and hiking trails, which can lead to more fatal outcomes, are needed.

Species distribution models (SDMs), which predict the potential habitats of a given species across space and time using environmental variables, utilize process-based and regression-based techniques or machine learning algorithms [18,19,20,21]. SDMs and GIS (geographic information system) have been utilized not only to reveal potential habitats by considering the ecological niches of venomous snakes but also to evaluate the risk of snakebite incidents. Yanez-Arenas et al. [22] analyzed the statistical associations between snakebite incidents and factors, such as human population density, marginalization index, and the proportion of the population without health insurance in Veracruz, Mexico, by combining generalized additive models (GAMs) and SDM results for six different venomous snake species. Melo Araújo et al. [23] utilized the SDM results of 17 venomous snake species and human population density in Maranhão, Brazil as predictor variables in a generalized linear model (GLM) to demonstrate a strong correlation between snakebite incidents and potential snake habitats. Yousefi et al. [24] overlaid the potential habitats of four different venomous snake species with human residential areas in Iran to identify high-risk areas for snakebite incidents. These studies [22,23,24] have all utilized SDMs to reveal the environmental characteristics affecting the potential distribution of venomous snakes as well as factors related to snakebite incidents at administrative levels. Although these studies hold significance at broad regional scales, studies evaluating the risk of snakebite incidents on walking paths, such as hiking trails, where direct accidents can occur, are lacking. In other words, to establish more realistic snakebite-accident prevention measures, research considering the scale at which people and snakes actually encounter each other is required.

The aim of this study was to evaluate the risk of snakebite accidents on hiking trails in South Korea. To achieve this, we used a random forest algorithm-based SDM to analyze the ecological characteristics and potential habitat of three *Gloydius* spp., representative venomous snakes in South Korea [25,26]. After examining the correlations between the metrics of snakebite accidents and SDM results, we combined information from the SDM results with hiking-trail spatial data to identify high-risk hiking trails in national parks in terms of *Gloydius* snakebite accidents. These findings can be utilized as valuable information for hikers, who may choose to exercise caution in potentially hazardous trail sections.

## 2. Materials and Methods

### 2.1. Study Area

**Figure 1:**
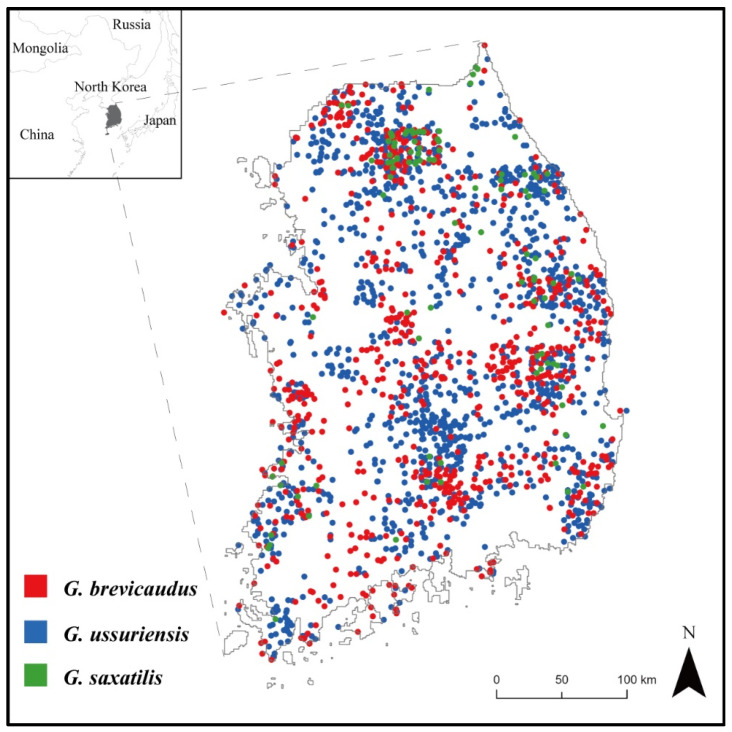
Presence locations of *Gloydius* spp. from the National Ecosystem Survey between the years 2006 and 2018 in South Korea (704 occurrence points for *G. brevicaudus*; 1519 for *G. ussuriensis*; and 100 for *G. saxatilis*).

**Figure 2:**
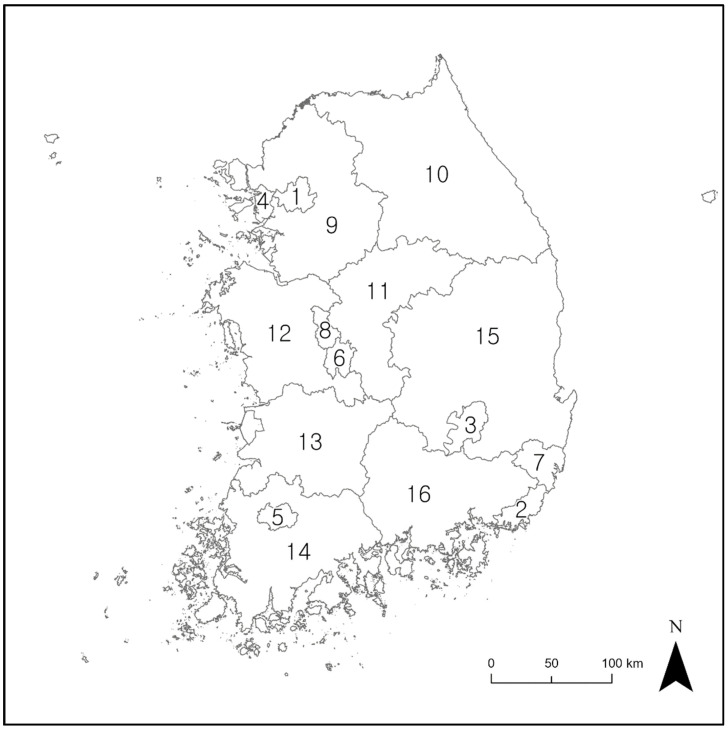
Municipal and provincial boundaries in Korea. The numbers are the IDs of the cities utilized in the study. 1: Seoul; 2: Busan; 3: Daegu; 4: Incheon; 5: Gwangju; 6: Daejeon; 7: Ulsan; 8: Sejong-si; 9: Gyeonggi-do; 10: Gangwon-do; 11: Chungcheongbuk-do; 12: Chungcheongnam-do; 13: Jeollabuk-do; 14: Jellanam-do; 15: Gyeongsangbuk-do; and 16: Gyeongsangnam-do.

The study area encompassed the inland regions of South Korea (latitude 33° to 39° N, longitude 124° to 130° E) and excluded island regions. This area covers approximately 100,000 km^2^ (Figure 1), has a population of about 51 million people, and there are 16 municipalities and provinces (Figure 2). Over 70% of the study area is covered by mountainous terrain, with the average altitude being approximately 500 m. In the eastern regions, a mountain range called Backdudaegan traverses the study area in a longitudinal direction. On the other hand, the western regions of the study area generally feature a mix of hilly and flat terrains. The climate is characterized by four distinct seasons: a clear and dry spring (March–May) and autumn (September–November); a cold and dry winter (December–February); and a hot and humid summer (June–August) [25]. The average temperatures in summer and winter are 24.1 °C and 0.6 °C, respectively. The annual precipitation is about 1234 mm, with the most precipitation occurring during the summer [25]. According to previous studies, *Gloydius* spp. can inhabit the entire study area [27], and are known to be active mainly from April to October and inactive during the winter months when they undergo hibernation [28,29].

### 2.2. Target Species

The three target *Gloydius* spp., short-tailed pit viper (*Gloydius brevicaudus*), red-tongue viper (*Gloydius ussuriensis*), and rock mamushi (*Gloydius saxatilis*), represent venomous snakes in South Korea and are primarily found in agricultural lands and mountainous terrain [30,31]. These three species were selected as the target species for this study. Over 60% of all snakebite incidents in South Korea are caused by *Gloydius* spp. [30,32]. Although South Korea is home to four venomous snake species, tiger keelback (*Rhabdophis tigrinus*) is less likely to cause fatal snakebites due to its having rear fangs located at the back of its mouth, in contrast to those of *Gloydius* spp. [33]. Furthermore, *R. tigrinus* primarily exhibits passive, defensive behavior that is not associated with snakebite accidents [34], which led to its exclusion from the group of target species in this study.

### 2.3. Data Collection

In this study, the location data of *Gloydius* spp. were obtained from the National Ecosystem Survey data provided by the Ecological Information Bank (https://nie-ecobank.kr, accessed on 19 April 2023) of the National Institute of Ecology in South Korea. The National Ecosystem Survey has been conducted every five years since 1986, and it involves ecological experts surveying the entire country to collect presence data on species’ habitats. For the statistical analysis in this study, we utilized the *Gloydius* spp. presence data collected from the National Ecosystem Survey between the years 2006 and 2018. We preprocessed the data to include only one point of each *Gloydius* spp. within a 30 m pixel. As a result, 704 points representing *G. brevicaudus*, 1519 points representing *G. ussuriensis*, and 100 points representing *G. saxatilis* were used in the SDMs (Figure 1).

To predict the potential habitats of *Gloydius* spp. with SDMs, we selected nine environmental variables based on previous studies [13,27,35,36,37,38] (Table 1). Elevation data (digital elevation model, DEM) with a 30 m resolution were obtained from the EarthExplorer (https://earthexplorer.usgs.gov, accessed on 19 April 2023). The collected DEM data were used to create two other predictors: slope and topographic position index (TPI) [39]. Four land-cover-related variables, representing bare land, herbaceous vegetation, shrubland, and forest percentage cover in 2015, were collected from the Land Cover Viewer [40]. Land-cover variables with a 100 m resolution, derived from satellite imagery, have a value between 0 and 100 depending on the level of the corresponding land cover within the satellite-image cell. We obtained two climate variables from WorldClim (https://www.worldclim.org, accessed on 19 April 2023) at a resolution of 2.5 arc minutes (or about 4.5 km): the annual average of the maximum and minimum temperatures between April and October, known to be the snakes’ active season, from 2014–2018, and precipitation for the same time period. Land-cover and climatic variables were resampled to a 30 m resolution using the bilinear technique in ArcGIS Pro 3.0.

To assess *Gloydius* spp. snakebite-risk areas, we collected hiking-trail data, major municipal and provincial boundaries, municipal and provincial human population data, and snakebite incident statistics. The hiking-trail data were obtained from the Forest Service [41] in polyline format and converted to raster data with a 30 m resolution for further analysis. Municipal boundaries in polygonal format were retrieved from GIS-Developer [42]. For human population data in Korea, we used data from Statistics Korea [43]. Snakebite accident statistics representing the number of snakebite patients in 2016 were collected from the online database of the Health Insurance Review and Assessment Service (HIRA) (Table 2) [44]. Furthermore, to determine the major hiking-trail sections in South Korea, we obtained national park boundary data from the Korea Database on Protected Areas (KDPA). Within South Korea, there are a total of 22 national parks; however, in this study, only 16 national parks were selected by excluding four marine and coastal parks, which were deemed unsuitable for *Gloydius* spp., as well as a park in Jeju Island and another with no hiking-trail data.

### 2.4. Species Distribution Modeling

In this study, we used the VisTrails platform’s extension tool, SAHM (Software for Assisted Habitat Modeling), which supports SDM analysis, and selected the highly accurate and stable random forest (RF) SDM. RF generates multiple decision trees for the relationship between environmental variables and target species locations using bootstrap resampling, allowing duplicates. The ensemble of decision trees generated without pruning can be used to create SDMs that integrate the responses of the target species’ occurrence to the given environmental variables through ensemble techniques, thereby reducing overfitting and maintaining the models’ predictive power [45,46,47]. For RF, the out-of-bag prediction error was minimized using the tuneRF function.

The data required to run RF-based SDMs in SAHM include presence and pseudo-absence data of the target species, environmental variables, and template data serving as the geographical reference for other datasets [48]. Presence data were obtained from the coordinates of each *Gloydius* spp. in the National Ecosystem Survey data. Pseudo-absence data were generated by creating 10,000 random points. Environmental variables including DEM, TPI, slope, landcover types, average of the maximum and minimum temperatures, and precipitation in tiff format were used. Boundary data of South Korea’s mainland in a 30 m resolution image (tiff format) were used as the template data.

To prevent multicollinearity among environmental variables, variables with absolute Pearson, Spearman, or Kendall correlation coefficients greater than 0.75 between each pair of variables and with low values of percent deviance explained were checked while building the SDMs. The percent deviance explained was obtained in a univariate generalized additive model (GAM) or generalized linear model (GLM), depending on which could be fit. High correlations (|*r*| > 0.75) were observed between DEM and the average of the maximum and minimum temperatures, as well as between herbaceous and shrubland cover. The average of the maximum and minimum temperatures and herbaceous cover showed higher values of deviance explained than DEM and shrubland cover. Therefore, to eliminate multicollinearity and improve the explanatory power of the final models, we excluded DEM and shrubland variables when building the SDMs.

Of the presence coordinates, 70% were used as training data, while the remaining 30% were used as test data. The performance of an SDM was evaluated using two metrics, the area under the receiving operating characteristic curve (AUC-ROC) and the true skill statistic (TSS). AUC-ROC is a metric that ranges between 0 and 1. A value of 1 signifies ideal differentiation between presence and absence, or pseudo-absence locations, while a score of 0.5 indicates that the discrimination by an SDM is no better than random guessing of the presence of a target species. A score below 0.5 indicates that the model’s performance is even worse than random. TSS is a threshold-dependent metric with little dependence on species prevalence [49]. TSS, which is equal to sensitivity plus specificity minus one, ranges from −1 to 1, with 1 indicating perfect performance, 0 indicating random performance, and negative values indicating worse than random predictions. We computed the TSS values with thresholds chosen by maximizing the sum of sensitivity and specificity of SDMs. We also applied ten-fold cross-validation to the training dataset for additional model evaluation with the AUC metric.

After evaluating SDMs based on the AUC-ROC and TSS, we generated binary maps in a 30 m resolution raster format to represent presence and absence of the target species. These maps were based on the threshold that maximized the sum of sensitivity and specificity of each SDM. In the binary map, 1 and 0 represent the presence and absence of *Gloydius* spp., respectively. Finally, the binary maps for each species of *Gloydius* were overlapped to create a comprehensive *Gloydius* spp. potential habitat map (GsppPHM), in which a value of 1 indicated that at least one species was present.

To evaluate the environmental variable importance of each *Gloydius* species’ SDM, we used the mean decrease Gini (MDG), a representative indicator of environmental variable importance in RF-based models. MDG indicates the extent to which each environmental variable influences the performance of the model. The higher the MDG value, the more important the environmental variable is for improving model performance [50]. The median MDG value for the three SDMs was used to select the top three environmental variables and present their response curves.

We next verified whether the snakebite risk areas derived from the GsppPHM had a statistically significant relationship with the instances of actual snakebite accidents in 16 regions in South Korea. The correlations between the number of snakebite patients per 10,000 people in each major region in 2016 and variables including the proportion of GsppPHM area, total area, human population density, hiking-trail area, and hiking-trail density of each region were analyzed using Spearman’s correlation test. Furthermore, the average of land cover (forest and cropland) scores was calculated to understand the land cover characteristics of the *Gloydius* spp. potential habitats in the GsppPHM. The risk levels of national parks were calculated and compared by analyzing the ratio of *Gloydius* spp. potential habitat areas to the total area of each national park. Additionally, the hiking trails within the national parks with the highest and lowest risk levels for *Gloydius* spp. snakebites were visualized to highlight the hiking trails with a higher risk of snakebite incidents.

## 3. Results

### 3.1. Model Performance Evaluation

The RF model for predicting *Gloydius* spp. habitats had a decent working performance (train and test AUC of 0.76 and 0.77 for *G. brevicaudus*, 0.80 and 0.81 for *G. ussuriensis*, and 0.81 and 0.82 for *G. saxatilis*; train and test TSS of 0.40 and 0.42 for *G. brevicaudus*, 0.45 and 0.46 for *G. ussuriensis*, and 0.47 and 0.41 for *G. saxatilis*). When evaluating model performance using a ten-fold cross-validation on the training dataset, all three species SDMs had good predictive power, with a mean test AUC of 0.76 (SD = 0.05) for *G. brevicaudus*, 0.80 (SD = 0.02) for *G. ussuriensis*, and 0.82 (SD = 0.11) for *G. saxatilis*. The main environmental variables evaluated in the *Gloydius* spp. SDMs were TPI, slope, and average of the maximum and minimum temperatures, followed by precipitation, forest cover, herbaceous cover, and bare cover. Among the predictors, TPI showed the highest MDG value in all SDMs for *Gloydius* spp. (Table 3).

**Figure 3:**
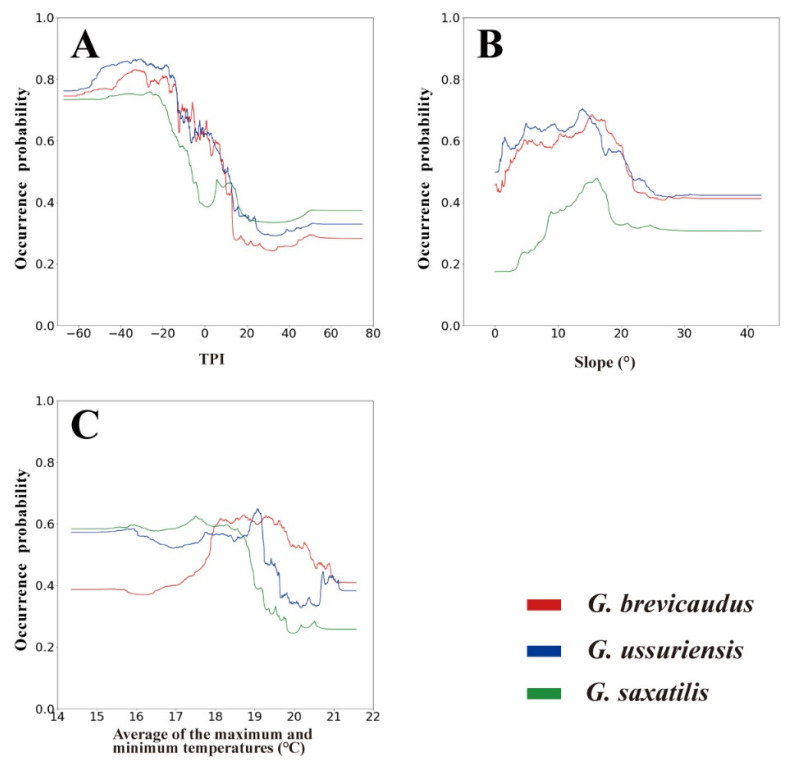
Species response curves of three major environmental variables: (**A**) TPI; (**B**) slope; and (**C**) average of the maximum and minimum temperatures.

According to the response curves for environmental variables, all species exhibited a strong preference for areas with TPI values below 0, indicative of valleys, and a slope of around 15° (Figure 3). These two variables generally exhibited similar patterns for all three species. *G. brevicaudus* showed preference for average of the maximum and minimum temperatures between ca. 18 and 20 °C, while *G. ussuriensis* and *G. saxatilis* favored temperatures of ca. 14–19 °C. In other words, the occurrence probability of *G. brevicaudus* sharply increased at 18 °C and remained high up to ca. 20 °C. In contrast, *G. ussuriensis* and *G. saxatilis* exhibited high occurrence probabilities below ca. 19 °C, with a sharp decrease in occurrence probability starting at ca. 19 °C.

### 3.2. Habitat Distribution of Gloydius spp. and Its Relationship with Snakebite Accidents

**Figure 4:**
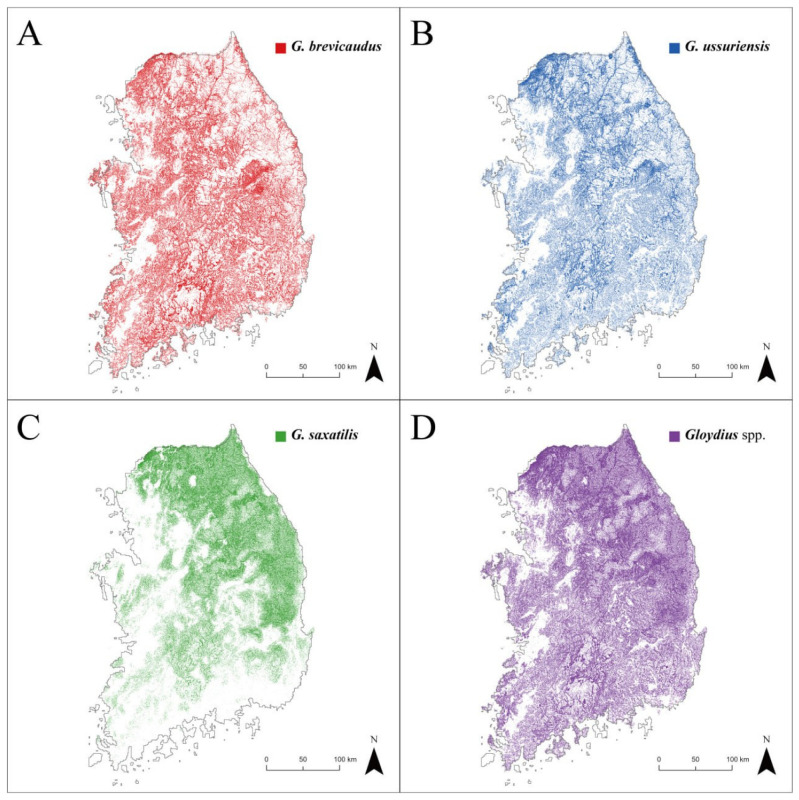
This map represents *Gloydius* spp. potential habitat. This is where snakebites can occur: (**A**) *G. brevicaudus*; (**B**) *G. ussuriensis*; (**C**) *G. saxatilis*; and (**D**) *Gloydius* spp. (i.e., GsppPHM).

The potential habitats of *Gloydius* spp. were visualized as binary maps based on the SDM results (Figure 4A–C). The potential habitats of *G. brevicaudus* (28,276 km^2^ and 31% of the study area) and *G. ussuriensis* (25,204 km^2^, 27%) were mainly in mountainous areas throughout the study area, with the exception of some high-altitude mountainous terrain in the east. Favorable habitats for *G. saxatilis* (23,585 km^2^, 26%) generally appeared in high-altitude mountainous areas, mostly in the eastern part of the study area. GsppPHM, the integrated potential presence map of *Gloydius* spp., revealed that 45% of the study area can potentially provide suitable habitats for *Gloydius* spp. (Figure 4D). According to the GsppPHM, suitable habitats for *Gloydius* spp. were more prevalent in the northeastern regions compared to the southwestern areas. Forested and cropland areas occupied an average of 56.38% and 13.38% of land cover, respectively, in areas identified as potential habitats for *Gloydius* spp. by the GsppPHM.

The number of snakebite accidents per 10,000 people was significantly correlated with the proportion of the GsppPHM area in major regions (Spearman’s rho = 0.638, *p* < 0.01). However, the other variables including total area, human population density, hiking-trail area, and hiking-trail density of major regions did not show any significant correlations with the number of snakebite accidents (*p* > 0.05).

### 3.3. Snakebite Risk Assessment in National Parks

**Figure 5:**
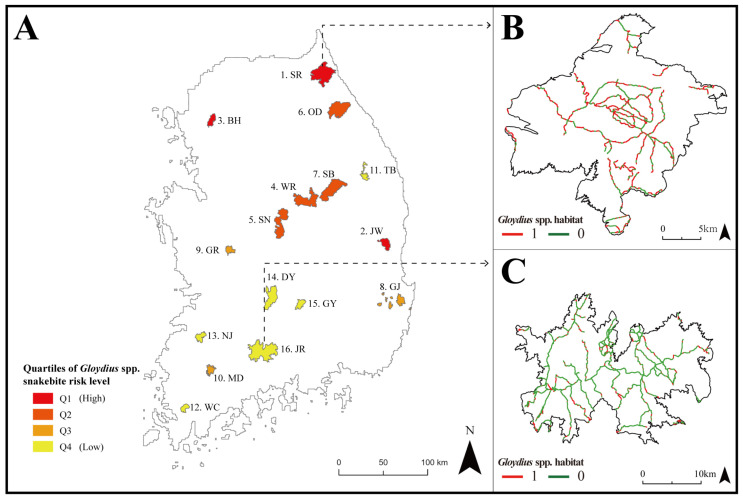
(**A**) Maps of *Gloydius* spp. snakebite risk of 16 national parks in South Korea (SR: Mt. Seorak; JW: Mt. Juwang; BH: Mt. Bukhan; WR: Mt. Worak; SN: Mt. Songni; OD: Mt. Odae; SB: Mt. Sobaek; GJ: Gyeongju; GR: Mt. Gyeryong; MD: Mt. Mudeung; TB: Mt. Taebaek; WC: Mt. Wolchul; NJ: Mt. Naejang; DY: Mt. Deokyu; GY: Mt. Gaya; and JR: Mt. Jiri); (**B**) hiking trails in SR with the highest snakebite risk; and (**C**) hiking trails in JR with the lowest snakebite risk among the national parks.

Since potential habitats identified by GsppPHM were primarily concentrated in the northeastern part of South Korea, national parks in these regions exhibited a high suitability for *Gloydius* spp., which translates to a high snakebite risk (Figure 5A). Among the national parks in South Korea, Mt. Seorak had the highest ratio of *Gloydius* spp. potential habitat area to total area (i.e., 57%) (Figure 5B). On the other hand, only 18% of the total area of Mt. Jiri (Figure 5C) was predicted to be potential habitat for *Gloydius* spp.

## 4. Discussion

### 4.1. Evaluating the Importance of Environmental Variables

In South Korea, the habitats of *Gloydius* spp. are primarily determined by altitude, which is correlated with the temperature and could be related to the avoidance of interspecific competition among *Gloydius* spp. [27]. Do et al. [51] have shown that changes in *Gloydius* spp. habitats in South Korea can be predicted based on various environmental factors, including altitude, land cover, and bioclimatic factors. In line with this study, they showed that altitude, temperature seasonality, and the maximum temperature of the warmest month are important environmental variables in determining potential *Gloydius* spp. habitats. Furthermore, the present study showed the importance of topographical conditions such as TPI and slope in the habitat selection of *Gloydius* spp.

Changes in temperature can affect habitat selection by influencing the hibernation, feeding, and reproductive activities of snakes [47,52,53]. Altitude is highly correlated with temperature [54] and therefore can influence snakes’ habitat selection. Do et al. [27] reported that *G. brevicaudus* prefers habitats located at low altitudes (94–311 m), while *G. ussuriensis* favors mid-altitude regions (108–407 m), and *G. saxatilis* prefers high altitudes (164–511 m). In other words, all three *Gloydius* spp. can be easily observed from about 100 m to 300 m of altitude, as this study identified the average of the maximum and minimum temperatures as an important predictor, which was highly correlated to altitude. This study also showed that all three species have a high occurrence rate in the common temperature range of 18 °C to 19 °C. In addition, the average of the maximum and minimum temperatures of preferred habitats decreased in the order of *G. brevicaudus*, *G. ussuriensis*, and *G. saxatilis*, which is consistent with the findings of Do et al. [27].

The results of this study revealed that TPI was the most significant factor in determining the potential habitats for *Gloydius* spp., which preferred areas near valley bottoms (Figure 3A). This preference is probably due to the fact that the prey of *Gloydius* spp., such as amphibians and fish, are often found near valley bottoms, indicating that the major activity range of *Gloydius* spp. is similar to the location of food resources [37,55,56,57]. Furthermore, slope was considered to be a factor limiting their habitat range, suggesting that *Gloydius* spp. prefer areas with moderate slopes [58]. These areas may optimize fitness by facilitating mobility, foraging, and burrow selection [59].

### 4.2. Snakebite Risk Assessment at a Regional Scale

The potential habitat of *Gloydius* spp. predicted by the SDM (GsppPHM) showed a significant correlation with snakebite accident statistics (*p* < 0.01). This result is similar to that of studies that demonstrated a significant relationship between snake habitat suitability predicted by SDMs and snakebite incidents [23,60]. Furthermore, both this study and a previous one [23] did not find a significant correlation between human population size or human population density and snakebite accidents. This finding suggests that snakebite accident risk is not simply higher in areas with high human population concentration, but rather largely influenced by the presence of people in the vicinity of habitats that present suitable topographic and bioclimatic features for snakes.

*Gloydius* spp. are mainly found in highly forested areas. However, according to Shin et al. [30], snakebite incidents occur most frequently in cropland areas (65%), followed by forested areas (28%). In forests, even when hikers encounter *Gloydius* spp., the probability of direct contact is low, as *Gloydius* spp. tend to hide in their shelters. In contrast, in croplands, relatively more snakebite incidents can occur because farmers can pass through crop areas that *Gloydius* spp. use as hiding places. Nevertheless, forests could be a more suitable habitat for *Gloydius* spp., possibly resulting in higher population sizes in forests. Therefore, like croplands, forests also require special caution, especially as recreation in forests continues to grow.

### 4.3. Risk Assessment of Gloydius spp. in National Parks

Among the 16 national parks in South Korea, Mt. Seorak contained the highest proportion of the GsppPHM (57% relative to the national park area). Mt. Seorak is a national park located in the mountainous region of Gangwon-do Province in the northeastern part of the study area. In contrast, Mt. Jiri, situated in the southern part of the study area in Jeollanam-do Province, had the lowest proportion of the GsppPHM (18% relative to the national park area). However, according to actual snakebite incident data (Table 2) [44], Gyeonggi-do Province had the highest number of snakebite cases, and Jeollanam-do Province had the highest rate of snakebite incidents per 10,000 people. These comparisons represent a discrepancy between the study results and statistical data.

Shin et al. [30] compared the literature (1970–2019) and medical data (2010–2019) related to snakebite incidents. According to the literature, Gangwon-do Province was the area with the highest concern for snakebite incidents, while the more recent medical data revealed Gyeonggi-do Province as the area of greatest concern for snakebite incidents, as shown in Table 2. Shin et al. [30] explained the difference between the two datasets as being due to regional biases related to human population density, snake populations, and accessibility to medical facilities. In this study, the correlation analysis between GsppPHM and snakebite statistical data resulted in a correlation coefficient of 0.638, indicating a relatively low correlation. This finding is presumed to be due to the regional bias explained by Shin et al. [30].

Mt. Seorak and Mt. Jiri are tourist attractions visited by about 3 million people annually [61]. In this study, 55% of the hiking-trail sections in Mt. Seorak matched with the GsppPHM, indicating that extra caution is needed when hiking in this area. In particular, Figure 5B reveals that all paths leading to the mountain summits in the national park have sections that coincide with the habitat of *Gloydius* spp. Therefore, visitors to Mt. Seorak should keep in mind the possibility of encountering *Gloydius* spp. On the other hand, only 17% of the hiking-trail sections in Mt. Jiri matched with the habitat of *Gloydius* spp. Figure 5C shows that more suitable habitats were found in the outskirts of Mt. Jiri, while fewer suitable habitats were discovered near the mountain summits. However, since suitable habitats were detected in some parts of all sections, caution is required, though not as much as in Mt. Seorak. Based on these data, hikers can set up cautionary zones for each section of the hiking trail, helping to prevent *Gloydius* spp. bite incidents.

### 4.4. Significance of the Research

Hiking is an easily accessible recreation that does not require special training or equipment [62]. Since major national parks in South Korea attract about 3 million annual visitors [61], many people could be exposed to the risk of snakebite accidents caused by *Gloydius* spp. The GsppPHM created in this study can be effectively utilized to identify the risk of snakebite accidents in advance when exploring additional hiking trails for visitors. Furthermore, it can provide scientific evidence for policy initiatives to establish medical and rescue systems in forested areas with a high risk of snakebite incidents at the local level.

The results of this study can also be used to efficiently identify hiking trails nationwide that require the implementation of snakebite accident-prevention measures, such as snake warning signs or snakebite response guidelines. This action is possible because, unlike most previous studies that assessed snakebite incident risk at the administrative district level, this study not only identified high-risk administrative districts but also determined risk at the hiking-trail level, which can be practically used for hiking-trail safety management.

### 4.5. Limitations of the Study and Future Research Directions

There are limitations to this study. First, in order to assess the risk of snakebite accidents in *Gloydius* spp., this study utilized species distribution models (SDMs) based on individual-level location data. However, in order to achieve a more accurate assessment of the risk of snakebite accidents, it is necessary to validate or improve the SDMs used in this study by utilizing density data of *Gloydius* spp. populations confirmed through field surveys. Second, this study focused only on the physical environmental characteristics of *Gloydius* spp. habitats in assessing the risk of snakebite accidents. However, it is necessary to consider various factors related to the spatial analysis of the risk of snakebite accidents after SDMs, such as the activity time, season, species competition, and the distance from surrounding medical facilities, as shown in previous studies [8,23,63]. Through these additional considerations, it will be possible to create a more sophisticated map of the risk of snakebite accidents and establish more strategic measures for preventing snakebite accidents. Lastly, the snakebite accident patient statistics provided by the Health Insurance Review and Assessment Service (HIRA) include not only the target *Gloydius* spp. but also *R*. *tigrinus*. However, considering that more than 60% of venomous snakebite accidents are caused by *Gloydius* spp., and since *R. tigrinus* has behavioral and morphological characteristics that are associated with infrequent accidents [33,34], this study focused on *Gloydius* spp. as the target species. Future research should utilize spatial data that accurately document the location and time of snakebite accidents for each venomous snake species to achieve a more precise analysis.

## 5. Conclusions

This study analyzed the potential habitats of *Gloydius* spp. using environmental variables and SDMs while also evaluating and validating the risk of snakebite incidents in South Korea. The results showed that 17% to 55% of the hiking trails of national parks in the study area coincide with *Gloydius* spp. potential habitats, indicating that a large number of hikers could be exposed to the risk of snakebite accidents caused by *Gloydius* spp. Furthermore, this study identified areas with a high risk of snakebite incidents at the hiking-trail level across inland South Korea. These results are expected to support the development of practical solutions to prevent injuries and fatalities due to snakebites among hikers. In the future, through both this study and a deeper understanding of the physiological and ecological characteristics of venomous snakes, as well as the epidemiology of their venom, it will be possible to achieve a more reliable assessment of snakebite-accident risk.

## Figures and Tables

**Table 1:** Environmental variables used for species distribution modeling.

Variable		Unit	Data Source (Reference)
Topological	Digital elevation model (DEM)	m	USGS’s EarthExplorer (https://earthexplorer.usgs.gov)
	Topographic position index (TPI)	-	Obtained from DEM
	Slope	° (degree)	
Land cover	Bare	%	Land Cover Viewer (https://lcviewer.vito.be/2015)
	Herbaceous		
	Shrubland		
	Forest		
Climatic	Average of the maximum and minimum temperatures	°C	WorldClim (https://www.worldclim.org)
	Precipitation	mm	

**Table 2:** Summary of snakebite accidents in 2016 [44] and related variables in 16 regions (i.e., cities and provinces) of South Korea [42,43].

ID	Region	Human Population (Million)	Area (km^2^)	Number of Snakebites	Number of Snakebites per 10,000 People
1	Seoul	9.93	605.24	78	0.08
2	Busan	3.50	732.46	28	0.08
3	Daegu	2.48	880.62	97	0.39
4	Incheon	2.94	350.81	45	0.15
5	Gwangju	1.47	498.01	73	0.50
6	Daejeon	1.51	539.16	85	0.56
7	Ulsan	1.17	1044.90	51	0.44
8	Sejong-si	0.24	464.86	14	0.58
9	Gyeonggi-do	12.71	10,045.13	528	0.42
10	Gangwon-do	1.55	16,590.84	316	2.04
11	Chungcheongbuk-do	1.59	7408.86	288	1.81
12	Chungcheongnam-do	2.10	8023.70	392	1.87
13	Jeollabuk-do	1.86	8024.87	267	1.43
14	Jeollanam-do	1.90	10,374.78	526	2.76
15	Gyeongsangbuk-do	2.70	18,901.51	569	2.11
16	Gyeongsangnam-do	3.37	9567.07	297	0.88

**Table 3:** The table below represents the mean decrease Gini (MDG) for environmental variables, showing their importance in descending order of median values for *Gloydius* spp.

Environmental Variable	*G. brevicaudus*	*G. ussuriensis*	*G. saxatilis*	Median
TPI	76.25	184.75	10.90	76.25
Slope	53.17	115.58	7.30	53.17
Average of the maximum and minimum temperatures	48.12	116.11	10.46	48.12
Precipitation	43.93	88.76	6.04	43.93
Forest cover	43.80	87.36	4.39	43.80
Herbaceous cover	44.75	76.49	3.94	44.75
Bare cover	11.96	24.51	1.09	11.96

## Data Availability

The data are available upon request from the corresponding author.
